# Oxygen sensors and neuronal adaptation to ischemia

**DOI:** 10.18632/oncotarget.13938

**Published:** 2016-12-15

**Authors:** Hugo H. Marti, Reiner Kunze

**Affiliations:** Institute of Physiology and Pathophysiology, Heidelberg University, Germany

**Keywords:** PHD, HIF, neuron, focal cerebral ischemia, adaptation, Neuroscience

Hypoxia research is in the focus, as three of its pioneers receive the Lasker Prize 2016 for their discovery of hypoxia-inducible transcription factors (HIFs) and prolyl-4-hydroxlase domain proteins (PHDs) as cellular oxygen sensors [1]. HIFs are heterodimers consisting of a labile α and a stable β unit. In the presence of molecular oxygen the α subunit is hydroxylated by PHDs leading to immediate proteasom al degradation.

In cerebral ischemia, brain hypoperfusion results in tissue hypoxia, combined with nutrient depletion. Given that neuronal energy generation primarily relies on oxidative glucose metabolism, and their striking susceptibility to excitotoxicity, neurons exhibit the highest vulnerability to ischemic stress among all cells of the central nervous system. PHDs control endogenous mechanisms in nerve cells that promote adaptation to hypoxia/ischemia. As PHDs have many additional targets beside HIFa subunits, it remains to be established whether PHD-mediated effects are HIF-dependent or rather HIF­independent.

Our major finding is that neuronal inactivation of PHD2 in mice improves recovery from cerebral ischemia [2, 3]. One could hypothesize that this protective effect is either a direct one or alternatively occurs in an indirect manner, whereby PHD2 deficient neurons produce factors that act in a paracrine fashion on neighboring endothelial cells, astrocytes or microglia. The resulting activation/ modulation of these cells could then in tum support survival of neurons. We and others have demonstrated experimental evidence for both - direct and indirect - protective mechanisms.

PHD2 inactivation in neurons resulted in HIF stabilization and subsequent transcriptional activation of erythropoietin and vascular endothelial growth factor (VEGF) [2, 3]. Both mediators are well known to be potent anti-apoptotic factors, which directly support neuronal survival. Accordingly, application of the PHD inhibitor FG-4497 strongly increased cell survival in pure neuronal cultures exposed to ischemic conditions [4]. In addition, neuronal PHD2 deletion resulted in increased expression of HIF-dependent glucose transporters and glycolytic enzymes [2]. A switch from oxidative to glycolytic metabolism might improve ischemic tolerance of PHD2 deficient neuronal cells by enabling oxygen-independent energy generation, and reduction of mitochondrial reactive oxygen species (ROS) production.

Recently, Quaegebeur et al. showed that genetic loss or inhibition ofPHD1 in mice improves brain tissue damage and sensorimotor deficit upon focal cerebral ischemia by reprogramming neuronal glucose metabolism [5]. PHD1 deficient neurons maintained energy production via oxidative phosphorylation in mitochondria, but preferentially metabolized glutamine instead of glucose. PHD1 inactivation in neurons further redirected glucose away from glycolysis into the oxidative pentose phosphate pathway (oxPPP), which preserved the redox state of glutathione, and thus improved ROS scavenging capacity in neurons upon ischemia-reoxygenation. Enhanced oxPPP flux in PHDl deficient neurons was HIF-independent, but clearly relied on up-regulation of TP53-inducible glycolysis and apoptosis regulator (TIGAR) via activation of the NF-κB transcription factor p65 [5].

In addition, neurons might conserve energy by reversible shut down of synaptic transmission, which is the main energy-consuming process in the brain. We previously showed that application of PHD inhibitors or neuron-specific deletion of PHD2 significantly decreased excitatory postsynaptic potentials and strongly impaired the induction of long-term potentiation [6]. Recently, Segura et al. confirmed our hypothesis that PHDs might control neuronal adaptation to hypoxia/ischemia at the level of synaptic activity [7]. Genetic inactivation of PHD2 but not of PHD1 in neurons or short-term exposure of neurons to hypoxia impaired dendritic spine maturation, caused spine regression, and reduced spine density. Remodeling of dendritic spines was accompanied with a decreased synaptic density, which caused attenuated excitatory but not inhibitory synaptic transmission. Interestingly, upon reoxygenation neurons showed a rapid morphological recovery of dendritic spines and restoration of synaptic transmission [7]. They further confirmed that the actin cross-linker filamin A (FLNA) is a target of PHD2-mediated proline hydroxylation, leading to rapid ubiquitination and proteasomal degradation of FLNA. Accordingly, PHD2 inactivation diminished proteasomal degradation of FLNA, thereby inducing its stabilization and accumulation in non-synaptic locations and increasing the ratio of FLNA to F-actin. The latter might rearrange the actin cytoskeleton to reduce the number of dendritic spines, synapses, and synaptic transmission [7]. Although a direct proof was not provided by the authors, their findings suggest that a rapid and reversible spine remodeling upon PHD2 inactivation may provide an endogenous adaptive mechanism that protects neurons against excitotoxic overstimulation during cerebral ischemia.

Alternatively, neuronal inactivation of PHD2 may result in better stroke outcome indirectly by targeting neighboring endothelial or glial cells. In fact, we found a clear angiogenic response in PHD2 deficient mice after onset of ischemia resulting in increased capillary density [3]. We also demonstrated modulatory effects on microglia and astrocytes, which lead to an attenuated inflammatory response and may thereby protect neurons from cell death [3].

Along this line, we and others demonstrated that small-molecule PHD inhibitors, applied systemically, improve brain tissue damage and functional deficits in rodent models of ischemic stroke [4]. However, we also provided evidence that combined genetic loss of neuronal HIF-1α and HIF-2α in mice attenuates tissue injury in the early acute phase after cerebral ischemia, although it clearly impairs functional recovery at later stages indicating that activation of the HIF system by PHD suppressors can also have adverse effects [8]. As related compounds from several companies are currently in clinical trials for treatment of renal anemia, this fact should be carefully considered, the more when therapeutic strategies based on the use of PHD inhibiting drugs for treatment of human patients suffering from ischemic stroke are developed.
